# The role of SLIT-ROBO signaling in proliferative diabetic retinopathy and retinal pigment epithelial cells

**Published:** 2011-06-08

**Authors:** Weiyan Zhou, Wenzhen Yu, Wankun Xie, Lvzhen Huang, Yongsheng Xu, Xiaoxin Li

**Affiliations:** 1 Key Laboratory of Vision Loss and Restoration, Ministry of Education, Department of Ophthalmology, Peking University People’s Hospital, Beijing, China; 2 Clinical Stem Cell Center, Peking University Third Hospital, Beijing, China

## Abstract

**Purpose:**

SLIT-ROBO signaling acts as a cue in neuronal guidance and plays a role in vasculogenesis and angiogenesis. The aim of this study is to explore the effects of robo1 and slit2 on the formation of fibrovascular membranes (FVMs) in samples from patients with proliferative diabetic retinopathy. The effects of advanced glycation end products (AGEs) on robo1 and slit2 expression in human retinal pigment epithelium (RPE) cells and the role of recombinant N-SLIT2 protein in human RPE cell regulation were investigated.

**Methods:**

Immunohistochemistry was performed to determine the presence and distribution of robo1 and slit2 in FVMs, and to confirm the effects of SLIT-ROBO signaling on FVM formation. The expression levels of robo1 and slit2 in RPE cells under basal and differential concentrations of AGEs were measured using real-time reverse transcription-polymerase chain reaction (RT–PCR), immunoblotting, or enzyme-linked immunosorbent assay. LY294002, an inhibitor of phosphoinositide 3-kinase (PI3K), was used to help determine the AGE signaling mechanism. Recombinant N-SLIT2 protein was used to study the effects of slit2 on RPE cells in vitro. Cell proliferation, migration, and cell cycling were assessed using an 3-(4,5-dimethylthiazol-2-yl)-2,5-diphenyltetrazolium bromide assay assay (MTT) assay, a Boyden chamber assay, and flow cytometry. Real-time RT–PCR and enzyme-linked immunosorbent assay were used to study vascular endothelial growth factor (*VEGF*) mRNA expression in and VEGF protein secretion from RPE cells.

**Results:**

Robo1 and Slit2 were expressed in FVMs in RPE cells coimmunostained for pancytokeratin. AGEs resulted in an increase in robo1 and slit2 levels in RPE cells, and inhibition of PI3K-blocked robo1 and slit2 expression. Recombinant N-SLIT2 protein increased proliferation, attachment, and migration of the RPE cells, and these cells demonstrated significant accumulation in the S phase compared to control cells. Furthermore, RPE cells treated with exogenous N-SLIT2 protein had higher levels of *VEGF* mRNA expression and VEGF protein secretion (p<0.05).

**Conclusions:**

Robo1 and slit2 may play a role in the formation of FVMs. The presence of AGEs increased levels of robo1 and slit2 in human RPE cells via signaling through the PI3K/Akt pathway. Recombinant N-SLIT2 protein increased the biologic activity of RPE cells, as well as the expression of *VEGF*. From these results, we may conclude that SLIT-ROBO signaling potentially contributes to the development of diabetic retinopathy.

## Introduction

Diabetic retinopathy is a leading cause of visual impairment and blindness [1]. Hyperglycemia plays an important role in the pathogenesis of diabetic complications by increasing the levels of advanced glycation end-products (AGEs), which are late products of nonenzymatic glycation [2]. The accumulation of AGEs appears to be a key factor in the development of diabetic retinopathy. AGEs are thought to promote many diabetic complications, including retinopathy, nephropathy, neuropathy, and cardiovascular disease [3,4].

The retinal vasculature is particularly vulnerable to damage in patients with diabetes; however, other types of cells in the retina are also at risk. Notable among these cells are retinal pigment epithelium (RPE) cells, which form part of the blood-retinal barrier and perform functions crucial to the maintenance of the visual system [5]. It was previously demonstrated that persistently high glucose levels may induce the proliferation of RPE cells. RPE cells play an important role in the pathogenesis of diabetic proliferative vitreoretinopathy by migrating through retinal breaks and contributing to the formation of proliferative membranes. In previous studies, RPE cells were demonstrated to secrete many different growth factors, and vascular endothelial growth factor (VEGF) was believed to play an important role in the neovascularization of the retina [6].

The SLIT-ROBO signaling cascade includes the Slit family of secreted proteins (Slit1, 2, and 3) and their corresponding receptors (Robo1, 2, 3, and 4). Although first characterized in neuronal development [7], Slit2 was recently found to interact with Robo1 to mediate repulsive cues in myogenesis [8], leukocyte chemotaxis [9], and endothelial cell migration. Slit2 has been widely identified in various human cancers, and the interaction between Slit2 and Robo1 is thought to induce tumor angiogenesis [10]. In addition, Slit2 has been identified as a regulator of adult lymphangiogenesis [11].

The full-length Slit2 protein is a 200 kDa secreted ligand that is cleaved into two smaller fragments, a 140 kDa N-terminal product (N-Slit2) and a 50–60 kDa C-terminal product (C-Slit2). N-Slit2 remains tightly bound to the cell membrane, whereas C-Slit2 is readily diffusible [12]. Little is known about the amino acid motifs involved in the interaction between N-Slit2 and the cell membrane, or whether further enzymatic processing might be necessary to generate additional N-terminal fragments in vivo. However, many studies have found that N-Slit2 binds to Robo, which is a single-pass transmembrane receptor [13].

In a previous study, we confirmed the presence of Robo1 and 4 expression in RPE cells. We indicated that Robo1 and 4 may play a role in the formation of FVMs in cells [14,15]. Robo1 and Robo4 may also be involved in retinal vasculogenesis and angiogenesis. In this study, we found that the FVMs stained positively for Robo1 and slit2 in RPE cells. We also investigated Robo1 and slit2 expression in cultured human RPE cells under different concentrations of AGEs or LY294002, an inhibitor of phosphoinositide 3-kinase (PI3K). Using recombinant human N-terminal Slit2 protein (N-Slit2), we were able to study the role of slit2 as a neuronal guidance cue in RPE cells.

## Methods

### Tissue samples

This study protocol was approved by the Ethics Committee of Peking University, and informed consent was obtained from all patients according to the World Medical Association Declaration of Helsinki. FVM specimens were surgically removed from the eyes of five patients with type 2 diabetes and proliferative diabetic retinopathy (PDR; 5 eyes), and who were undergoing pars plana vitrectomy with membrane peeling. Study patients consisted of three males and two females, with ages ranging from 50 to 70 years (mean age of 61.6±8.29 years). The duration of diabetes pathogenesis ranged from 6 to 25 years (mean duration 16±7.78 years). The five FVM specimens were fixed in a test tube containing 4% paraformaldehyde (PFA), and were subsequently embedded in optimum cutting temperature compound for immunohistochemistry.

### Cell culture and reagents

Human RPE cells (D407 cell line) were obtained from the American Tissue Culture Collection (Manassas, VA) and were cultured in Dulbecco’s Modified Eagle Media (DMEM; Gibco, Invitrogen, Grand Island, NY) with 10% fetal bovine serum (FBS, Gibco, Invitrogen), 100 units/ml penicillin, and 100 μg/ml streptomycin (Sigma, St. Louis, MO) at 37 °C under 5% CO_2_ and 95% humidity. RPE cells were plated in 6-well culture dishes and were used for experiments at 80%–90% confluence. The cells were incubated in fresh serum-free medium for 24 h before use in experiments. For culture under conditions for AGEs (Biovison, Linda Vista Avenue, CA), cells were treated using unmodified BSA or AGEs (10, 20, 50, 100 ug/ml). To assess PI3K/Akt activity, the pharmacological inhibitor LY294002 (Sigma, St. Louis, MO) was added to the culture medium at a final concentration of 10 μM for 30 min before treatment with AGE (20 ug/ml). To culture cells in the presence of recombinant N-SLIT2 protein (PeproTech, Rocky Hill, NJ), cells were treated at final concentrations of 0.1, 1, 10, and 100 ng/ml protein.

### RNA isolation

Total RNA was isolated using Trizol reagent (Invitrogen, Carlsbad, CA) according to the manufacturer’s instructions. After being washed with 75% ethanol, the final RNA extracts were eluted in 20 μl distilled diethyl pyrocarbonate–treated water. The concentration and purity of RNA were measured using a spectrophotometer. All RNA preparations had an OD260:OD280 ratio of 1.9–2.0.

### Real-time PCR

Retinal RNA (2 μg) was converted into cDNA in a total reaction volume of 25 ul, containing 1 ug oligo (dT)15, 5 μl Moloney Murine Leukemia Virus (M-MLV) 5× reaction buffer, 1.25 ul deoxy-ribonucleoside triphosphate mix (dNTPs), 25 U recombinant RNasin RNase inhibitor, and 200 U M-MLV reverse transcriptase. The mixture was incubated for 60 min at 42 °C, and reverse transcription was terminated by incubation of the solution at 95 °C for 5 min [14]. Real-time PCR assays were performed according to the manufacturer’s instructions (Fermentas, Burlington, Canada). The absorbance were measured at 562 nm on a plate reader. Use the standard curve to determine the protein concentration of each sample. Sequence-specific primers for β-actin (*ACTB*), human Robo1, human slit2, or human *VEGF* (Table 1) were used. Real-time PCR was performed using IQ Supermix (Bio-Rad, Hercules, CA), with each 20 μl reaction mixture containing 2 μl cDNA, 7.2 μl sterilized water, 10 μl SYBR Green Real-time PCR Master Mix (2×), and 0.8 μl of each primer (10 μM). Amplification was performed in 96-well plates using an iCycler iQ real-time detection system (Bio-Rad). Thermocycling conditions consisted of 3 min at 95 °C to activate the iTaq DNA polymerase, 35 cycles of 20 s each, a 95 °C denaturation step, a 15 s 61 °C (*VEGF* and *SLIT2*) or 63 °C (*ACTB* and *Robo1*) annealing step, and a 15 s 72 °C extension step. *Robo1*, *slit2*, and *VEGF* were normalized to *ACTB* expression and DNA levels were calculated using the following equation: Fold change=2^−ΔΔct^.

**Table 1:** Gene subtype oligonucleotide primers.

**Gene subtype**	**Oligonucleotide primers (5′-3′)**	**Size (bp)**
*ACTB*	F: CTTAGTTGCGTTACACCCTT	144
	R: CCTTCACCGTTCCAGTTT	
human *Robo1*	F: GGAAGAAGACGAAGCCGACAT	104
	R:TCTCCAGGTCCCCAACACTG	
human *Slit2*	F:CACCTCGTACAGCCGCACTT	107
	R:TGTGGACCGCTGAGGAGCAA	
human *VEGF*	F: AGTTCCACCACCAAACATGC	110
	R: TGAAGGGACACAACGACACA	

### Western blot analysis

Cells were washed three times in ice-cold phosphate-buffered saline (PBS, 4 °C, 8.00 g NaCl, 0.20 g KCl, 0.24 g KH_2_PO_4_, and 1.44 g Na_2_HPO_4_ in 1 l distilled water, pH 7.4) every 5 min at room temperature and prepared using protein extraction and protease inhibitor kits (Pierce, Rockford, IL). Cell lysates were cleared by centrifugation at 12,000× g at 4 °C. The supernatant was collected, and the protein content of each lysate was measured using a BCA Protein Assay Kit (Tianlai Shengwu Jishu, Tianlai, China) according to the manufacturer’s instructions. The standard and sample were added into a microplate well. It was incubated at 37 °C for 30 min. Equal amounts (20 μg) of protein were electrophoresed on 10% sodium dodecyl sulfate (SDS) polyacrylamide gel and analyzed by immunoblotting. Primary antibodies used include anti-Robo1 (1:500; Abcam, Cambridge, UK, Cat No. ab7279) and β-actin (1:1000; Boster, Wuhan, China). Membranes were washed and incubated with peroxidase-conjugated secondary antibodies (1:6000; Boster, China), and proteins were visualized using enhanced chemiluminescence western blotting detection reagents (Pierce) according to the manufacturer’s recommendations. Robo1 band densities were normalized to each β-actin internal control. All immunoblots were repeated three times, and qualitatively similar results were obtained across blots.

### Measurement of enzyme-linked immunosorbent assay

Cell supernatants were analyzed for slit2 (Cusabio Biotech Co., Wuhan, China) and VEGF (R&D Systems, Minneapolis, MN) levels using commercially available enzyme-linked immunosorbent assay (ELISA) kits. Conditioned media were collected after 24 h of incubation in either AGEs or N-SLIT2 protein, and the media was centrifuged and stored at −70 °C until analysis. Measurements were conducted according to the manufacturer’s instructions, and all samples were assayed in triplicate and mean values calculated.

### Immunohistochemistry

Membrane tissues were snap-frozen and 6 μm sections were cut. Thawed tissue sections were air dried, placed in 4% PFA for 20 min for fixation, washed with PBS, and blocked with 10% normal goat serum for 1 h at 37 °C. Next, 1:100 anti-Robo1 polyclonal antibody (Cat No. ab7279; Abcam, Cambridge, UK) and 1:200 anti-slit2 polyclonal antibody (Cat No. AB 5701; Millipore, Temecula, CA) with 1:100 anticytokeratin antibody (Santa Cruz, Santa Cruz, CA) was applied to the tissue sections at 4 °C overnight and incubated for 1 h at 37 °C with 1:100 fluorescein isothiocyanate– and tetramethylrhodamine isothiocyanate–conjugated goat antirabbit and goat antimouse secondary antibodies (Santa Cruz), respectively. Following incubation, slides were washed, and cell nuclei were stained with 4’, 6’-diamino-2-phenylindole (DAPI). Images were acquired with a fluorescence microscope equipped with a digital camera. For each of the immunostaining procedures, negative controls included omission of the primary antibody and use of an irrelevant polyclonal or isotype-matched monoclonal primary antibody. In all cases, negative controls showed only faint, insignificant staining.

### In vitro cell proliferation assay

To assess cell proliferation, a 3-[4,5-dimethylthiazol-2-yl]-2,5-diphenyltetrazolium bromide (MTT; Roche, Molecular Biochemicals, Mannheim, Germany) assay was used. Briefly, RPE cells were plated at a density of 2×10^3^ cells per well in 96-well culture plates. After attachment, the culture medium was changed to DMEM containing 10% FBS, and the cells were incubated for 24 h. After reaching 80% confluence, the cells were starved with DMEM containing 1% FBS for 6 h and then treated with various concentrations of N-SLIT2 protein. After 24 h, MTT was added to the culture medium and the cells were incubated for an additional 4 h. Formazan crystals that formed were then dissolved by the addition of dimethyl sulfoxide (100 μl/well). Absorbance at 570 nm was measured using an ELISA plate reader (Dynatech Medica, Guernsey, UK) [14]. Each experimental condition was performed in quadruplicate wells, and each plate was repeated three times.

### Cell attachment assay

Plates (96 well) coated with 1.25 μg/ml fibronectin in 100 μl PBS were incubated overnight at 4 °C. RPE cells (1×10^4^) treated with various concentrations of N-SLIT2 protein (10, 100 ng/ml) were trypsinized, added to each well, and permitted to attach for 6 h [14]. The cells were then washed gently twice with PBS, and 150 μl fresh medium with MTT was added to each well. Absorbance was measured using an ELISA plate reader at 570 nm. Three different wells were used to detect cell attachment, and all experiments were repeated three times.

### Cell migration

The cell migration assay was performed as described previously [14]. Briefly, 2×10^4^ cells were placed in the upper chamber in a final volume of 200 μl serum-free medium. Next, 10% FBS with recombinant N-SLIT2 protein (10, 100 ng/ml) was placed in the bottom chamber for a final volume of 600 μl. All migration assays were conducted for 4 h at 37 °C. At the end of the assay, the cells were fixed in 4% PFA and stained with DAPI for 15 min. The remaining cells were removed with a cotton bud, and the membrane was imaged. The number of cells from five random fields of view was counted.

### Flow cytometry

RPE cells (1×10^6^) were seeded in 6-well plates and treated with recombinant N-SLIT2 protein (10, 100 ng/ml) for 48 h. Cells were detached using ethylene diamine tetraacetic acid (EDTA), washed in ice-cold PBS (4 °C), and treated with the BD Cycletest™ Plus DNA Reagent Kit (Becton Dickinson) according to the manufacturer’s protocol. Samples were analyzed using a FACS Caliber cytometer (Becton Dickinson). Three samples were used per experiment, and each experiment was repeated.

### Statistical evaluation

All data were evaluated for normality of distribution. Statistical differences were evaluated using ANOVA followed by the Student–Newman–Keuls test for multiple comparisons. p<0.05 was considered statistically significant. All data are presented as mean±standard deviation (SD).

## Results

### Immunohistochemical detection of robo1 and slit2 in FVMs

**Figure 1:**
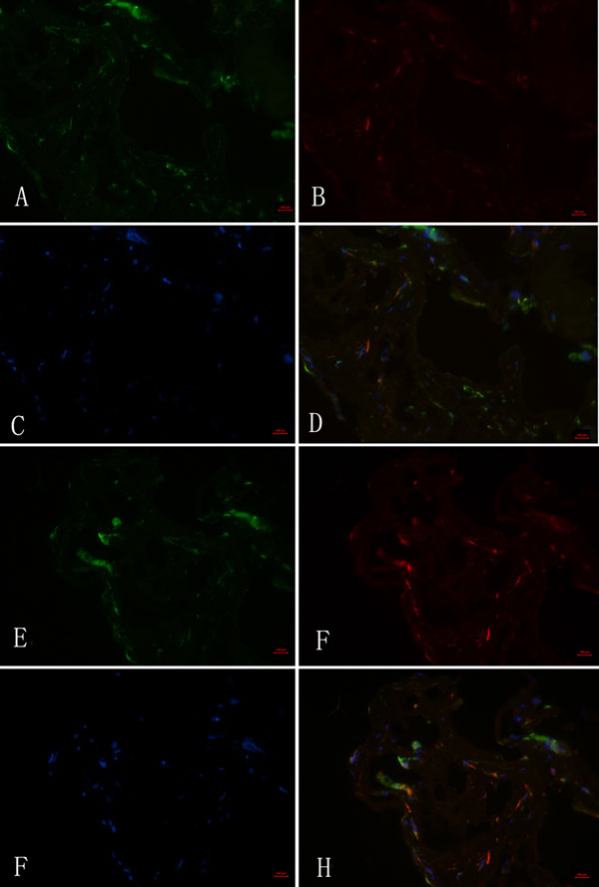
Robo1 and slit2 expression in fibrovascular membranes from a 66-year-old patient with a 15-year history of diabetes. **A**, **B**, **E**, **F**: Immunofluorescent staining shows Robo1-positive (**A**), slit2-positive (**E**), and cytokeratin-positive (**B**, **F**) staining in fibrovascular membranes (FVMs). **C**, **D**, **G**, **H**: Cell nuclei were stained with 4’, 6’ -diamino-2-phenylindole (DAPI; **C**, **G**), and the colocalization of Robo1 and pancytokeratin (**D**), and slit2 and cytokeratin (**H**) are also shown. Bar graphs denote 100 μm.

To investigate the presence and distribution of robo1 and slit2 in FVMs, we stained sections using an antirobo1 antibody (Figure 1A), an antislit2 antibody (Figure 1E), and an anticytokeratin antibody (RPE cell marker, Figure 1B,F). Detection with the robo1 and slit2 antibodies demonstrated that these proteins were expressed in FVMs. Additionally, we determined that robo1 and slit2 were coexpressed with cytokeratin, which is a marker of RPE cells.

### Effects of advanced glycation end-products and LY294002 on expressions of *robo1* and *slit2* mRNA in human retinal pigment epithelium cells

**Figure 2:**
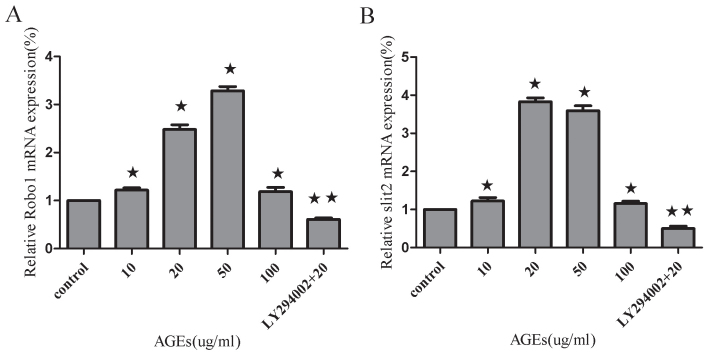
Effects of advanced glycation end-products and LY294002 on Robo1 and slit2 mRNA levels in human retinal pigment epithelium cells as measured by real-time RT–PCR. **A**: Robo1 expression in human retinal pigment epithelium (RPE) cells was significantly increased at the mRNA (mRNA) level after a 24 h treatment of advanced glycation end products (AGEs) as measured by real-time RT–PCR. LY294002 (10 uM) blocks the expression of robo1 under conditions of AGEs (20 ug/ml). **B**: Slit2 expression in human RPE cells was also increased at the mRNA level after AGEs. The expression of slit2 in response to AGEs (20 ug/ml) decreased after treating cells with LY294002 (10 uM) versus treating the cells with AGEs (20 ug/ml) alone. Values provided are the mean±SD of three independent experiments. Asterisks denote values significantly different between the treated and control groups (p<0.05). Double asterisks denote values significantly different between the LY294002 (10 uM) group and the AGEs (20 ug/ml) group (p<0.01). The relative expression level of the control group cell was set to 1.

We observed that the presence of AGEs (10, 20, 50, or 100 ug/ml) increased robo1 and *slit2* mRNA levels in human RPE cells measured using real-time RT–PCR (Figure 2). These results are representative of three experiments with similar outcomes. AGEs at a concentration of 50 ug/ml induced the strongest robo1 mRNA expression level, with a 3.29 fold increase in *robo1* mRNA expression compared to the control group (p<0.01). It was also determined that LY294002 (10 uM) blocked the expression of robo1 under AGEs (20 ug/ml; p<0.01; Figure 2A). We also demonstrated that mRNA levels of *slit2* in human RPE cells increased after exposure to AGEs. However, the highest expression of *slit2* mRNA was seen with an AGE concentration of 20 ug/ml. There was a 3.83 fold increase in *slit2* mRNA expression compared to control (p<0.01). *Slit2* expression secondary to AGEs exposure (20 ug/ml) was also blocked using LY294002 (10 uM; p<0.01; Figure 2B). In addition, we found that 100 ng/ml AGEs induced a slight upregulation of *robo1* and *slit2* mRNA (p<0.05).

### Effects of advanced glycation end-products and LY294002 on Robo1 protein expression in human retinal pigment epithelium cells

**Figure 3:**
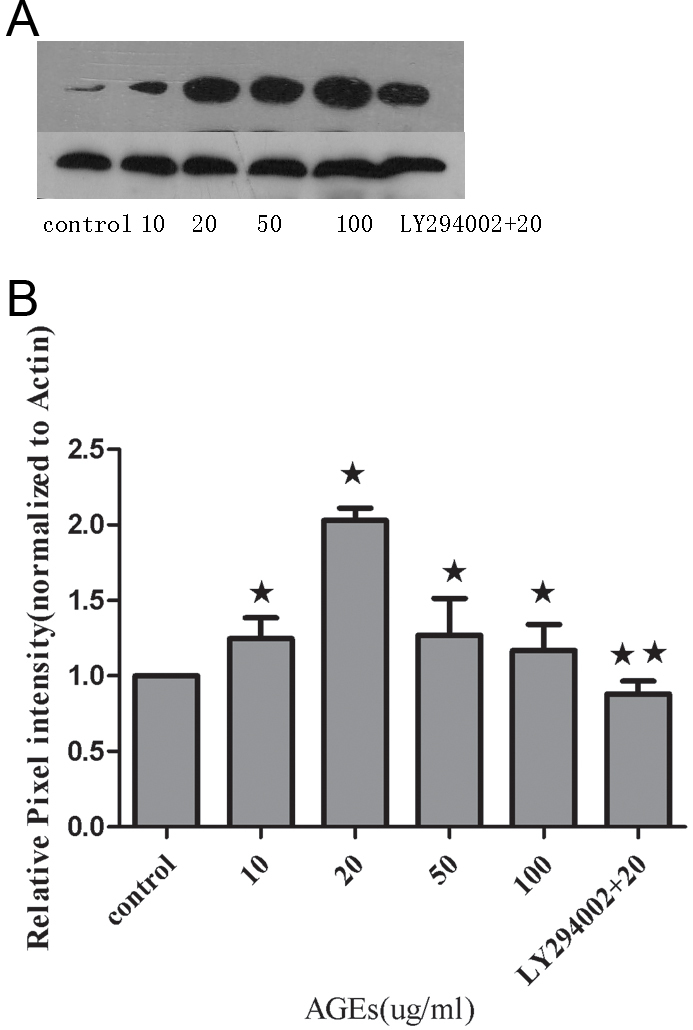
Protein expression of Robo1 in human retinal pigment epithelium cells was measured by immunoblotting with normalization to β-actin expression in retinal pigment epithelium cells. **A**: A representative photograph of the immunoblot analysis for Robo1 expression in human retinal pigment epithelium (RPE) cells. **B:** Relative Robo1 protein levels between the control group, advanced glycation end-products (AGEs) group, and LY294002-treated group. Values provided are mean±SD of three independent experiments. Asterisks denote values significantly different between the treated group and control group (p<0.05). Double asterisks denote values significantly different between the LY294002 (10 uM) group and AGEs (20 ug/ml) group (p<0.01).The pixel intensity of the control group was set to 1.

We next evaluated whether Robo1 protein levels are also regulated in AGE-treated RPE cells. Immunoblotting was performed using total cell lysates from RPE cells cultured with various concentrations of AGEs for 24 h. Robo1 protein expression in response to 20 ug/ml AGEs demonstrated a 2.03-fold elevation compared to the control group (p<0.01). The AGEs (20 ug/ml) group was then treated with LY294002, and robo1 expression was demonstrated to decrease to that of treatment with single AGEs (20 ug/ml; p<0.01; Figure 3).

### Effects of advanced glycation end-products and LY294002 on secretion of slit2 protein in human retinal pigment epithelium cells

**Figure 4:**
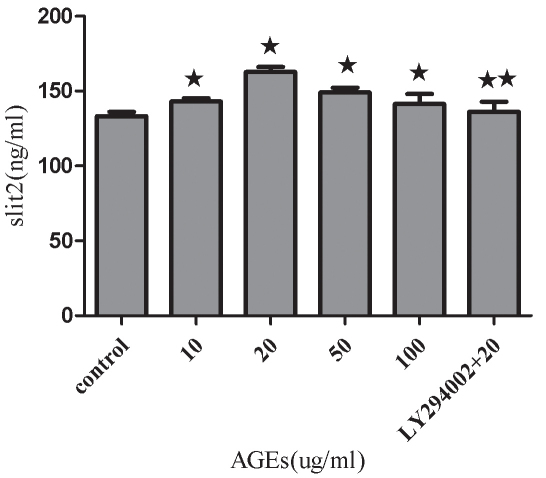
Effects of increasing concentrations of advanced glycation end-products and LY294002 on the secretion of slit2 protein in human retinal pigment epithelium cells. Cells were treated with increasing concentrations of advanced glycation end-products (AGEs) ranging from 10 to 100 ug/ml and LY294002 for 24 h in serum-free media. Slit2 protein levels in human retinal pigment epithelium (RPE) cells were measured using an enzyme-linked immunosorbent assay, and it was demonstrated that AGEs resulted in an increase in slit2 secretion. LY294002 was shown to decrease the expression of slit2 protein under AGE treatment conditions (20 ug/ml). Values are reported as the mean±SD from three independent experiments. Asterisks denote values significantly different between the treated group and control group (p<0.05). Double asterisks denote values significantly different between the LY294002 (10 uM) group and the AGEs treatment (20 ug/ml) group (p<0.01).

Slit2 protein secretion by RPE cells was increased in the presence of various concentrations of AGEs as measured by ELISA. Cells treated with a concentration of 20 ug/ml AGEs induced the highest levels of slit2 protein (p<0.01). When the concentration of AGEs rose to 50 and 100 ug/ml, slit2 protein expression was decreased compared to 20 ug/ml AGEs (p<0.05). LY294002 was determined to block the expression of slit2 protein under conditions of AGE treatment (20 ug/ml; p<0.01; Figure 4).

### Effects of recombinant N-SLIT2 protein on the proliferation of human retinal pigment epithelium cells

**Figure 5:**
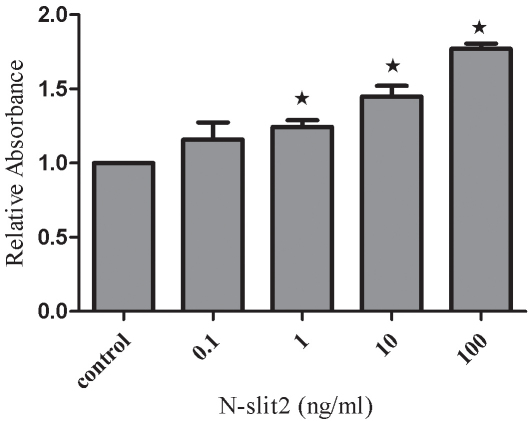
Effects of recombinant N-SLIT2 protein on human retinal pigment epithelium cell proliferation. Retinal pigment epithelium (RPE) cell proliferation was measured using an MTT assay at 24 h. Values reported are mean±SD from three independent experiments. Asterisks denote values significantly different from those of cells treated with recombinant N-SLIT2 protein compared to control cells (p<0.05).

We initially performed experiments to evaluate whether recombinant N-SLIT2 protein had any effect on RPE cell proliferation. Cells were incubated with N-SLIT2 protein at concentrations of 0.1, 1, 10, or 100 ng/ml for 24 h. Among the various concentrations of N-SLIT2 protein tested, N-SLIT2 protein at a concentration of 10 ng/ml or 100 ng/ml was observed to significantly increase RPE cell proliferation compared to the control group. These data suggest that N-slit2 may significantly induce RPE cell proliferation until a threshold concentration of 100 ng/ml. N-SLIT2 protein (100 ng/ml) was determined to increase cell proliferation by 77% after 24 h compared to control cell proliferation (p<0.05; Figure 5).

### N-SLIT2 protein regulates cell attachment and migration

**Figure 6:**
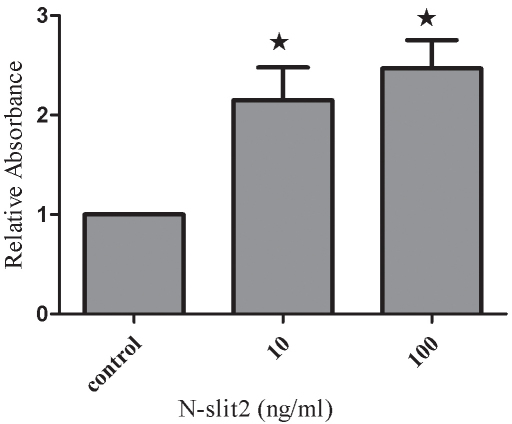
Effects of recombinant N-SLIT2 protein on the attachment of human retinal pigment epithelium cells. Cell attachment was assessed after 6 h of incubation and subsequent MTT assay. Values are reported as mean±SD of four independent experiments. Asterisks denote values significantly different from those of cells treated with recombinant N-SLIT2 protein compared to control cells (p<0.01). The absorbance of the control cells was set to 1.

**Figure 7:**
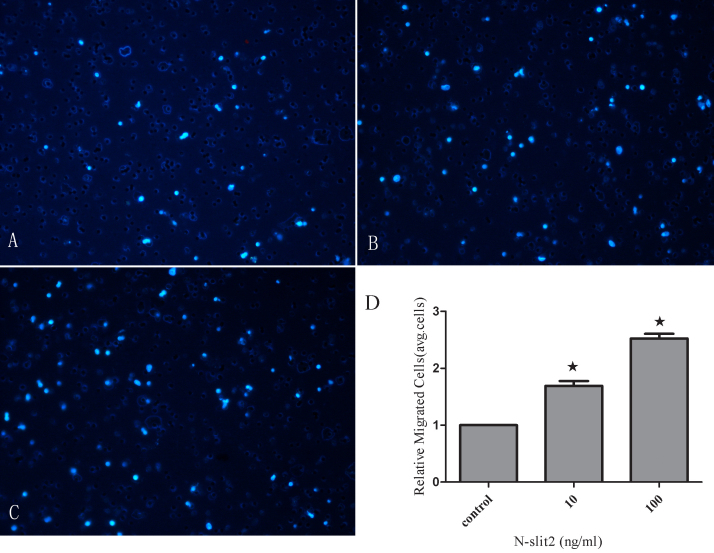
Effects of recombinant N-SLIT2 protein on human retinal pigment epithelium cell migration. The migratory activity of cells was estimated based on the number of cells that had traveled through the filter of the chamber. **A**: Control human retinal pigment epithelium migration. **B**: Migration of cells treated with recombinant N-SLIT2 protein (10 ng/ml). **C**: Migration of cells treated with recombinant N-SLIT2 protein (100 ng/ml). **D**: Relative migration of cells in the control and recombinant N-SLIT2 protein groups. Values are reported as the mean±SD from three independent experiments. The results demonstrate that the number of migratory cells in the recombinant N-SLIT2 protein group was greater than that in the control group (**D**, * p<0.01). Migration of cells in the control group was set to 1.

In the cell attachment assay, N-SLIT2 protein (10 ng/ml) treatment increased the attachment capacity of RPE cells by 2.15 fold (p<0.01; Figure 6) after 6 h compared to the control group, and 100 ng/ml N-SLIT2 protein increased the attachment by 2.47 fold compared to the control group (p<0.01; Figure 6). Next, we explored the role of N-SLIT2 in the migration of RPE cells using a modified Boyden chamber in which RPE cells migrated through a porous membrane. As shown in Figure 7, the mean number of migrated cells in the N-SLIT2 protein–treated RPE cells (10, 100 ng/ml) was significantly higher than the mean number of migrated control cells (p<0.01). Pretreatment of RPE cells with 10 ng/ml N-SLIT2 resulted in a 1.69 fold increase in attachment compared to the control group, and 100 ng/ml N-SLIT2 protein increased attachment by 2.52 fold (Figure 7).

### N-SLIT2 protein regulates cell cycle

**Figure 8:**
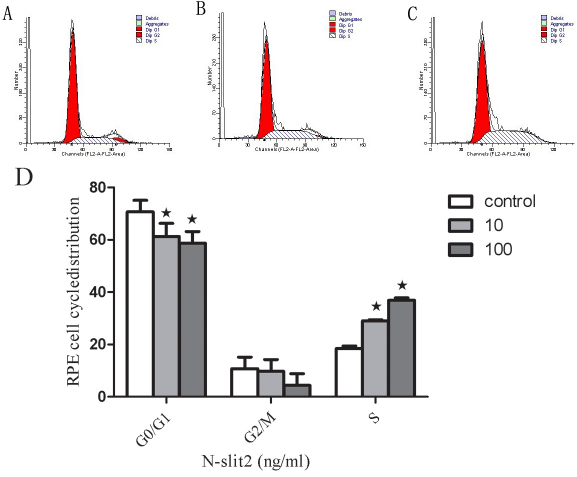
Effects of recombinant N-SLIT2 protein on the cell cycles in human retinal pigment epithelium cells. **A**: Cell cycle of control retinal pigment epithelium (RPE) cells. **B**: Cell cycle of RPE cells treated with recombinant N-SLIT2 protein (10 ng/ml). **C**: Cell cycle of RPE cells treated with recombinant N-SLIT2 protein (100 ng/ml). **D**: Data from the RPE cell cycle distribution of the control group and the recombinant N-SLIT2 protein group. Flow cytometric analysis demonstrates the effects of recombinant N-SLIT2 protein on the human RPE cell cycle. The x-axis represents fluorescence intensity on a logarithmic scale and the y-axis represents the number of events. The results show that the fraction of cells in the G1 phase has decreased and the proportion of cells in the S phase has increased in the presence of recombinant N-SLIT2 protein. Values are the mean±SD from three independent experiments. The proportion of G0/G1, G2, and S phase cells was demonstrated to be decreased in RPE cells treated with recombinant N-SLIT2 protein compared to control cells (**D**, *p<0.05).

As shown in Figure 8, the recombinant N-SLIT2 protein resulted in a significant reduction of RPE cells in the G_0_/G_1_ phase and promoted an accumulation of cells in the S phase compared to controls. Of the cells treated with recombinant N-SLIT2 protein, 61.31% and 58.7% were in the G_1_ phase when treated with 10 and 100 ng/ml, respectively, compared to 70.73% of the control group in the G_1_ phase (p<0.05; Figure 8). Moreover, in RPE cells treated with recombinant N-SLIT2 protein, 29.01% and 36.91% were in the S phase of the cell cycle, respectively, compared to 18.51% of cells in the control group (p<0.05; Figure 8). There was no significant difference between the treated and control groups in the number of cells in the G_2_/M phase (Figure 8).

### Effects of recombinant N-SLIT2 protein on the expression of VEGF in human retinal pigment epithelial cells

**Figure 9:**
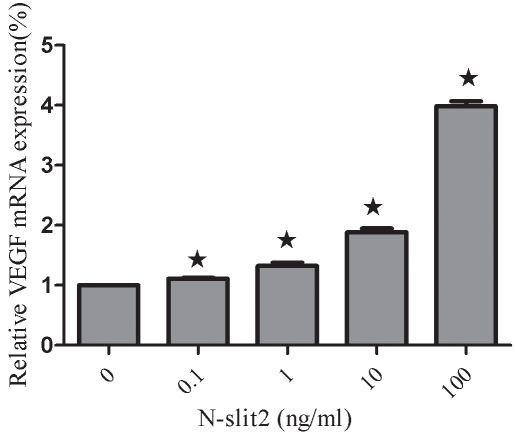
Effects of recombinant N-SLIT2 protein on vascular endothelial growth factor mRNA levels in human retinal pigment epithelium cells. Vascular endothelial growth factor (*VEGF*) mRNA expression in human retinal pigment epithelium (RPE) cells was significantly increased with increasing concentrations of recombinant N-SLIT2 protein as measured by real-time RT–PCR 24 h after recombinant N-SLIT2 administration. Values are reported as the mean±SD from three independent experiments. Asterisks denote values significantly different between the treatment group and control group (p<0.05). The relative expression level of the control group cell was set to 1.

**Figure 10:**
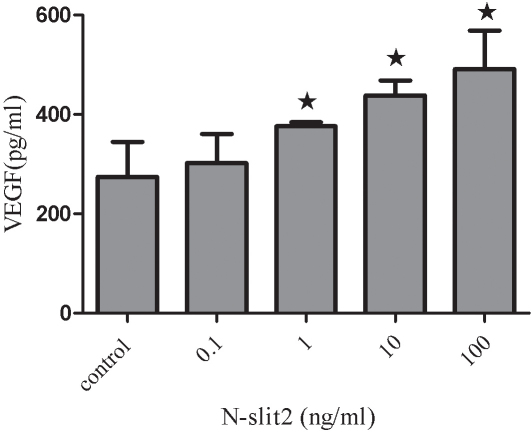
Effects of recombinant N-SLIT2 protein on vascular endothelial growth factor protein levels in human retinal pigment epithelium cells. Vascular endothelial growth factor (VEGF) protein levels were measured by enzyme-linked immunosorbent assay, and it was determined that VEGF secretion from human retinal pigment epithelium (RPE) cells increased in a direct manner with increasing administration of recombinant N-SLIT2 protein (0.1, 1, 10, 100 ng/ml). Values are reported as the mean±SD from three independent experiments. Asterisks denote values significantly different between the treatment group and control group (p<0.05).

We evaluated whether VEGF levels might be affected in RPE cells treated with N-SLIT2 protein. *VEGF* mRNA was measured using real-time RT–PCR, and the result is shown in Figure 9 as a representative experiment of three with similar outcomes. We found that the *VEGF* expression in N-SLIT2-treated cells was significantly increased compared to control cells, and *VEGF* mRNA expression levels increased with increasing recombinant N-SLIT2 protein concentrations (p<0.05). Recombinant N-SLIT2 protein at a concentration of 100 ng/ml increased *VEGF* mRNA levels to 3.98 fold greater than the control group (p<0.05). We next evaluated whether VEGF protein levels were also regulated by recombinant N-SLIT2 protein VEGF protein secretion by RPE cells, as measured by ELISA. Expression of *VEGF* was found to increase as N-SLIT2 protein concentrations rose, and a concentration of 100 ng/ml showed the strongest increase in *VEGF* mRNA (Figure 10) compared to controls (p<0.05).

## Discussion

Slit-Robo signaling was first demonstrated as an extracellular cue to guide axonal growth. However, signaling via this cascade was also demonstrated to regulate blood vessel branching. In our previous studies, we found for the first time that Robo1 and robo4 mRNA and protein were expressed in the FVMs of human eyes in patients with PDR. The results of dual-color immunofluorescence analyses of FVMs also showed positive Robo1 and robo4 immunostaining in the fibrous-like tissue and neovascular endothelial cells. We also observed that Robo1 and robo4 are expressed in choroid – retina endothelial (RF/6A) and RPE cells. These signaling molecules are thought to be involved in retinal vasculogenesis and angiogenesis [14-16]. Importantly, PDR is caused in part by increased retinal vascular permeability, new retinal vessel development, and/or loss of retinal capillaries [17]. Our current study is the first to examine whether Slit-Robo signaling, and specifically the effects of recombinant N-SLIT2 protein, may play a role in human RPE cells under diabetic conditions.

The fact that RPE cells contribute to the PDR membrane has been confirmed by ultrastructural investigation [18]. Furthermore, patients with PDR typically have RPE cells that are contained (5%–20%) in combined traction with rhegmatogenous retinal detachment membrane [19]. These observations all indicate that RPE cells migrate through the retinal breaks to access the PDR membrane and may contribute to PDR progression by secreting angiogenic factors. Moreover, the RPE is also known to represent the earliest pathological change in the diabetic retina [20]. In our study, we demonstrated for the first time that Robo1 and slit2 are expressed in the FVMs of patients’ eyes with PDR. The results of dual-color immunofluorescence analyses of FVMs showed positive Robo1 and slit2 staining in RPE cells. Robo1 and slit2 staining partially colocalized with cytokeratin. It is our hypothesis that RPE cells may autosecrete slit2, which then binds the single-pass transmembrane receptor, Robo1, under conditions of diabetic retinopathy. Following this idea, SLIT-ROBO signaling may actively participate in the progression of diabetic retinopathy. These observations suggest that Robo1 and slit2 may be important in the formation of FVMs.

Most studies have used human primary cells or RPE cells as an in vitro study model. One of the drawbacks of using primary RPE cells is that this model provides a limited number of cells and may lack a consistent cellular background. However, an engineered cell line may be able to overcome this problem while still maintaining the morphological and functional characteristics of primary cells [21]. Therefore, we used the D407 cell line for the majority of our studies. It is well known that hyperglycemia is a major risk factor for the development and progression of diabetic retinopathy. A central question in the understanding of this disorder has been the mechanism through which hyperglycemia results in diabetic retinopathy. Although chronic hyperglycemia is known to affect gene expression in several different tissues and cells [22], changes in Robo1 and slit2 levels were not examined in the present study. AGEs are believed to be the late products of nonenzymatic glycation linked with the development of diabetic retinopathy. In this study, we incubated RPE cells in varying concentrations of AGEs and used real-time RT–PCR, immunoblotting, and ELISA to demonstrate that AGEs promote the upregulation in Robo1 and slit2 expression. AGEs supplemented at a final concentration of 20 ug/ml or 50 ug/ml induced the strongest Robo1 and slit2 expression. Interestingly, when the AGE concentration rose to 100 ug/ml, Robo1 and slit2 expression decreased compared to that seen with 20 ug/ml AGEs. These results are similar to a previously published study where a concentration of 20 μg/ml AGEs resulted in increased cell numbers; however, it was postulated that higher concentrations of AGEs may cause a reduced rate of cell division, an increased frequency of apoptosis, or both [23]. This suggests to us that robo1 and slit2 may be involved in the pathologic action of AGE. PI3K and its downstream target molecule, serine-threonine kinase (Akt), are important in the regulation of several different cellular responses [24]. Inhibition of this pathway may be considered as a promising method to block RPE cell response in proliferative retinopathies [25]. We studied the PI3K/Akt signaling pathway using LY294002, a well known inhibitor of PI3K. We found that LY294002 is able to block the effects of AGEs (20 ug/ml) on robo1 and slit2 expression in RPE cells (Figure 2, Figure 3, and Figure 4). These findings comprise, to the best of our knowledge, the first data suggesting a role for the PI3K/Akt pathway in mediating AGE activity through the expression of robo1 and slit2.

It is known that adult RPE cells are quiescent and differentiated, and they reside in the G0 phase of the cell cycle. In diseases such as PDR [26], proliferative vitreoretinopathy [27], and age-related macular degeneration [28], attachment of cells to the retinal surface, spreading of cells along the surface, and cellular migration with subsequent membrane formation [29,30] are the initial pathophysiological events. In this study, human RPE cells were incubated in recombinant N-SLIT2 protein (0.1, 1, 10, 100 ng/ml) to evaluate the effects of N-SLIT2 on RPE cell proliferation. We found that the N-SLIT2 protein significantly increases RPE cell proliferation compared to the control group. Additionally, we observed that recombinant N-SLIT2 protein may play a role in many of the processes involved in the early events of pathogenesis. For example, we found that a concentration of 10 and 100 ng/ml N-SLIT2 protein increases both the attachment and migration of RPE cells. At the same time, we also found that 10 and 100 ng/ml recombinant N-SLIT2 protein causes significant reductions in the numbers of cells in the G_0_/G_1_ phase and an accumulation of cells in the S phase compared to the control group. Taken together, these results suggest to us that recombinant N-SLIT2 protein is important in initiating the pathogenesis of diabetic retinopathy.

VEGF is generally considered to be a key angiogenic factor in the neovascularization that occurs during PDR [31]. The hyperglycemia that is typically present in human and experimental diabetic retinopathy has been linked to enhanced VEGF activity [32,33]. Based on the results shown in Figure 9 and Figure 10, it is clear that the recombinant N-SLIT2 protein promotes a dose-dependent increase in *VEGF* mRNA expression as well as VEGF secretion by human RPE cells. The goal of the present study is to examine whether the recombinant N-SLIT2 protein, a neuronal guidance receptor, may enhance VEGF secretion and thus play a role in VEGF-induced angiogenesis.

In summary, our study indicates that Slit-Robo signaling may play a role in the formation of FVMs. Increasing concentrations of AGEs resulted in increased Robo1 and slit2 expression in RPE cells. The PI3K inhibitor, LY294002, is able to block the expression of robo1 and slit2 under diabetic conditions. The recombinant N-SLIT2 protein also results in the increased proliferation, attachment, and migration of RPE cells; it also results in an increased number of RPE cells in the S phase compared to the control group, the recombinant N-SLIT2 protein treatment of RPE cells increases *VEGF* mRNA expression and VEGF secretion. Although preliminary, all of these results suggest to us that Slit-Robo signaling may be involved in diabetic retinopathy. Nevertheless, additional studies are required to elucidate the precise role of Slit-Robo signaling in the development of diabetic retinopathy and to examine its potential role as a therapeutic target.
